# The Detection of Cancer Epigenetic Traces in Cell-Free DNA

**DOI:** 10.3389/fonc.2021.662094

**Published:** 2021-04-29

**Authors:** Anastasia P. Koval, Konstantin A. Blagodatskikh, Nikolay E. Kushlinskii, Dmitry S. Shcherbo

**Affiliations:** ^1^ Institute of Translational Medicine, Pirogov Russian National Research Medical University, Moscow, Russia; ^2^ Laboratory of Clinical Biochemistry, N.N. Blokhin Cancer Research Medical Center of Oncology, Moscow, Russia

**Keywords:** cell-free DNA, liquid biopsy, epigenetics, biomarkers, cancer diagnostics and screening

## Abstract

Nucleic acid fragments found in blood circulation originate mostly from dying cells and carry signs pointing to specific features of the parental cell types. Deciphering these clues may be transformative for numerous research and clinical applications but strongly depends on the development and implementation of robust analytical methods. Remarkable progress has been achieved in the reliable detection of sequence alterations in cell-free DNA while decoding epigenetic information from methylation and fragmentation patterns requires more sophisticated approaches. This review discusses the currently available strategies for detecting and analyzing the epigenetic marks in the liquid biopsies.

## Introduction

The pools of circulating nucleic acids found in biological fluids have been extensively studied in recent decades, primarily due to the minimally invasive sampling procedures that promise a number of apparent practical benefits. First, an opportunity to identify molecular changes that underlie pathological processes associated with cell death occurring in distant tissues from a simple blood draw. Second, real-time monitoring of the alterations through a sequential sampling series without disturbing the pathological foci. Third, cell-free nucleic acids in blood circulation are believed to represent a cumulative pool of fragments originating from different sources in the body, allowing for a snapshot of alterations that occur at various locations with increased cell turnover or active release (1–3), a key feature for cancer investigation in the light of tumor heterogeneity.

Currently, these fundamental properties of cell-free DNA (cfDNA) analysis are translated to several applications in clinical oncology, generally termed as liquid biopsy, namely, molecular tumor profiling (4–6), therapy response monitoring (7–9), minimal residual disease (MRD) and recurrence detection (10–12), as well as early cancer diagnostics (representing a highly desirable but still elusive output) (13). Most of these clinical aims can be partially achieved through careful analysis of somatic mutations present in the tumor fraction of cfDNA (circulating tumor DNA, ctDNA) (14–16). The recent technological advances in the field fostered ultra-sensitive variant detection methods that nevertheless should be thoroughly validated in extensive independent studies before being fully adopted for reliable clinical use (17–20).

Aside from sequence alterations, tumorigenesis is characterized by early occurring and further extensive genome-wide epigenetic changes that tune expression programs in favor of tumor-specific phenotypes (21–23). Variations in CpG methylation, histone modifications, and chromatin remodeling occur mainly in a patterned and cell-type-specific fashion, making these processes vast sources of attractive cancer biomarkers. However, in contrast to sequence alterations, these changes are not encoded in DNA sequence and have to be extracted from cell-free DNA with indirect methods. Here we are going to briefly summarize currently available approaches and future prospects of cfDNA epigenetic analysis for cancer diagnostics.

## Biology of Circulating DNA

The current understanding of cfDNA biology (3, 24, 25) and particularly epigenetics (26) has been recently reviewed elsewhere. However, to further discuss analytical methods, we have to emphasize some critical points. It is believed that the bulk of cell-free nucleic acids is formed as a byproduct during the course of cell death scenarios (27). The contribution of different cell types is still a matter of debate, as is the balance between active and passive release mechanisms and the possible functional roles being attributed to cfDNA subfractions by some researchers (3, 24, 25). Nevertheless, there seems to be a consensus on the high complexity of cfDNA pools found in biological fluids, which makes the tracing of sequence alterations and epigenetic marks found in cfDNA back to the cells of origin a challenging task.

The exact paths that DNA molecules may follow on their ways from the nucleus and mitochondria to the blood are not fully understood. The median cfDNA length of only ~165 bp reported in most studies (28–30) suggests that high molecular weight genomic DNA encounters nucleases during the shedding to the bloodstream. Surprisingly, despite being present in higher copy numbers in cells, mitochondrial DNA seems less accessible in plasma (31). This could be explained by the circular structure of mtDNA or its even higher fragmentation (32) due to the lack of nucleosomal structure and histone-mediated protection from nucleases in contrast to nuclear DNA. At the same time, mtDNA levels in plasma may provide clues on some pathological conditions, including cancer (33–37). Recent research highlights the roles of extrachromosomal DNA (ecDNA) in tumor progression (38–40). Often highly amplified and oncogene-enriched ecDNA molecules are detectable in blood plasma and may serve as an additional source of novel biomarkers after the development of appropriate analytical methods (41–43).

The turnover of cfDNA in blood seems to be rapid, with a half-life range of approximately 0,5-3 hours and a bias towards slower elimination of protein-bound DNA (44–46). If these estimates are true, any cfDNA test represents a nearly real-time snapshot of the cellular genomes. The sequence alterations and aberrant methylation that occurred in tumors seem to be apparently stable in ctDNA, while the inconsistency in alteration detection between tissue specimens and circulating DNA is most likely explained by the biological complexity of cancers and technical constraints (47–50). However, further studies are required to support the stability of tumor-specific alterations in cfDNA, especially aberrant methylation.

## Principles of Detection and Challenges

**Figure 1:**
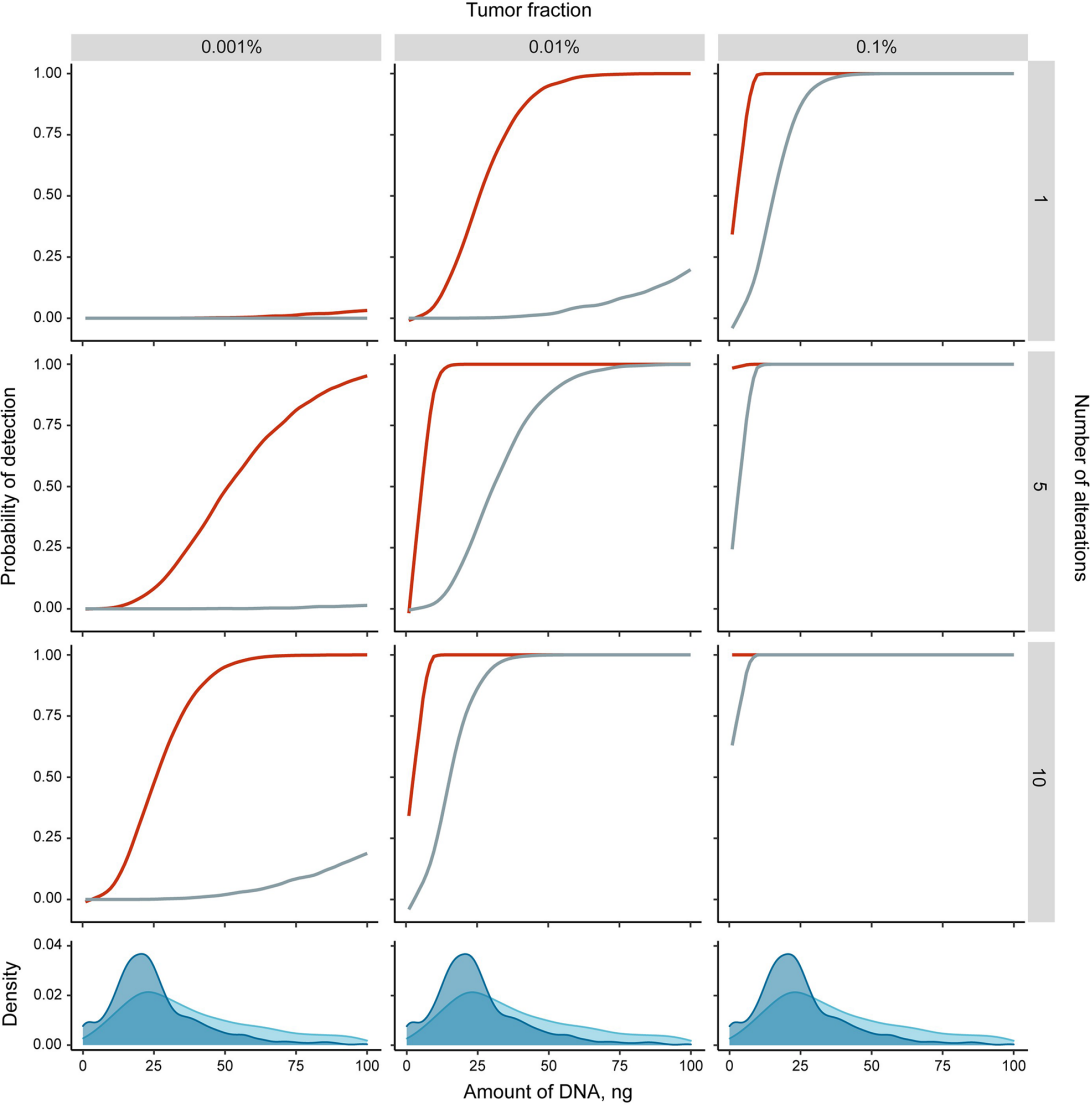
Theoretical simulations of the detection probability of single tumor-specific alteration (point mutation (grey) or differentially methylated region (DMR, red)) in cfDNA. Monte Carlo simulations were performed in R (see **Supplementary Text** for details) with the following assumptions: a DMR is represented by 6 co-methylated CpG sites (51); at least 5 observations of any alteration is required to classify the sample as cancer positive (adjustment for DNA loss during sample preparation and detection errors intrinsic to analytical methods); alterations are independent. Density plots at the bottom panels show the distributions of sampled cfDNA amounts in the four recent studies (16, 30, 52, 53) [3286 samples total, of which 1716 were from healthy individuals (blue) and 1570 from cancer patients (light blue)].

The detection of tumor-specific changes is the key for most cfDNA applications in oncology, but it is complicated by individual variability of ctDNA fraction, heterogeneity of cancer genomes, and the limited amount of cfDNA usually available for analysis. Our simulations suggest that the reliable detection of a single point mutation is theoretically limited by a ctDNA fraction of 0,1% for typically sampled cfDNA amounts (**Figure 1**). In contrast, the detection of aberrant cytosine methylation may result in higher overall sensitivity due to the tendency of the densely clustered CpG sites to share the same methylation state at least at the distances of up to 50-100 nucleotides (2, 54–57). Consequently, tumor-derived DNA fragments from differentially methylated regions (DMRs) technically carry a number of point epimutations in contrast to single nucleotide substitutions. It increases the theoretical probability of tumor DNA detection with epigenetic methylation-based assays (**Figure 1**). Moreover, the ensemble nature of epigenetic changes in cancer leads to a patterned structure of DMRs across the genome that further multiplicates the number of tumor-specific markers available for detection (22, 58).

The rate of cfDNA release from the tumor, or cfDNA shedding, is another crucial factor for successful cancer detection. The ability to release cfDNA varies significantly among individuals and cancer types (16). The proposed explanations of the variability in ctDNA shedding kinetics among tumor types include distinct anatomical features (blood-brain barrier, organ capsules, vascularization), mitotic/cell death rates, metabolic characteristics, and the predominant cell death mechanisms (59, 60). Tumor clinicopathological parameters significantly associated with the increased ctDNA shedding are higher tumor stage, nodal metastases, solid adenocarcinoma pattern, tumor necrosis, larger primary tumor diameter or volume, and frequent mitosis in tissue specimens (61). The detection of ctDNA was considerably higher in certain malignancies like pancreatic, ovarian, colorectal, bladder, gastroesophageal, breast, melanoma, hepatocellular, and head and neck cancers. In contrast, ctDNA was detected in only <50% of the primary brain, renal, prostate, or thyroid cancers (62). A recently proposed mathematical model of cfDNA shedding suggests that the probability of a false negative for a particular actionable mutation clonally present in tumors with diameters of 1 and 2 cm is 82 and 9.3%, respectively (at 99% specificity) (63).

Taking the above-mentioned into consideration, minimizing DNA loss and suppressing analytical errors during all steps of analysis is crucial for robust identification of lower tumor fractions. The recovery and purity of cfDNA during the preanalytical step are strongly dependent on careful protocol selection and validation (64–67). For instance, undesirable lymphocyte gDNA contamination can be avoided by either minimizing time before plasma separation to less than 4 hours or storage in stabilizer-containing tubes (68). It has been shown that size-selection in favor of shorter fragments (90-150 bp range) can enrich tumor fraction and consequently increase the sensitivity of upstream variant detection (28), which logically implies that the opposite is also true and suggests that the DNA size distribution should be controlled during sample processing.

### Methylation-Based Approach

Methylation of cytosines serves as an additional layer of instructive annotations for hard-coded genetic information in a cell-type-specific manner (69). Genome-wide cataloging of methylation patterns typical for normal and pathological tissues may result in a reference atlas that could be used for tissue-of-origin deconvolution from cfDNA methylation and revealing tumor localization (1, 2, 51, 70–72). However, the heterogeneity of cell types comprising most tissues increases noise and complicates identifying common specific patterns (1). An opposite idea, finding a universal methylation signature shared by multiple cancer types, may result in the development of pan-cancer early detection tests (52). Notably, the methylation profiles of cfDNA fraction derived from normal tissues must be taken into account as an inevitable background when determining DMRs. Despite the experimental wide-range approaches driving the field of liquid biopsy research, the analysis of limited marker sets is still more feasible in a practical setting due to lower costs and more straightforward interpretation. The genome-wide analysis of tumor-specific methylation, however, may yield novel candidates for designing narrow assays based on PCR or targeted sequencing of a small number of DMRs (73), thus expanding the selection of epigenetic cancer biomarkers such as methylation of *SHOX2* and *SEPT9* loci among others (74–77).

The two primary forms of modified cytosine in the human genome are 5-methylcytosine (5mC) and 5-hydroxymethylcytosine (5hmC). The latter can be considered not only as a product of 5mC oxidation by TET dioxygenases during demethylation but also as an independent epigenetic mark of the loci being activated (78–81). Nevertheless, these modifications are not directly detectable by most of the widespread sequencing or PCR-based methods, which makes some type of 5mC/5hmC-discriminating modification or enrichment a necessary step in the protocols. Chemical bisulfite conversion of unmethylated C to U underlies most PCR-based methods, methylation arrays, and sequencing approaches (82). Further development resulted in bisulfite-based oxBS-seq and TAB-seq protocols that differentiate between 5mC and 5hmC (83, 84). However, the related DNA loss of up to 90% (85–87) is a crucial obstacle for the analysis of low-input cfDNA samples. Another drawback intrinsic to the tactic of unmodified cytosine to uracil conversion is the reduced complexity of the output DNA sequence, which perplexes probe design and bioinformatic analysis (88, 89). The recently proposed conversion methods rely on enzymatic or combined treatments, which are reported to be less disruptive for DNA integrity. Particularly, TET dioxygenases can convert 5mC and 5hmC further to 5-carboxylcytosine (90), which can be either converted to dihydrouracils in TAPS protocol or protected from APOBEC-mediated deamination of C to U in EM-seq (91–93). Both methods with some modifications allow for 5mC and 5hmC discrimination.

Alternative approaches to methylation analysis rely on affinity enrichment, for instance, with 5mC-antibodies as proposed in cfMeDIP-seq (94, 95). Notably, the direct comparison suggests higher sensitivity of this method compared to sequence variant analysis (96), and further studies confirmed its utility for the detection of low-shedding renal (97) and intracranial tumors (98). In the 5hmC-Seal hydroxymethylcytosine, residues are selectively labeled with biotin and further captured on avidin beads (99, 100). The feasibility of this method for cancer detection was demonstrated in several studies (101–103). Moreover, a combination of cfMeDIP-seq and 5hm-Seal for simultaneous 5mC and 5hmC profiling in pancreatic cancer improved the prediction accuracy (104). MBD-seq takes advantage of the methyl-binding proteins such as MBD2 to capture methylated DNA (105–107). The protocol has been modified for low DNA input and showed performance similar to bisulfite sequencing (108), but its utility for cfDNA analysis has not been thoroughly evaluated. In contrast to most conversion strategies, affinity-based enrichment for methylated sequences may be more cost-effective at a whole-genome scale in applications where single-base resolution is not required since it allows to sequence predominantly methylated regions. On the other hand, it does not selectively target regions of interest and requires specific statistical tools capable of analyzing enrichment data.

### Fragmentation-Based Approach

Genome-wide cfDNA sequencing revealed a biased fragmentation pattern that correlates with chromatin organization levels from nucleosomal occupancy to high order 3D structure (109–111). These observations can be explained by more efficient cleavage of accessible DNA in open-chromatin regions in contrast to better-protected protein-bound DNA in a closed inactive conformation. In turn, the changes in chromatin accessibility reflect shifts in transcription regulation (112), thus indirectly connecting cfDNA fragmentation features to gene expression programs in parental cells. Moreover, for reasons that are still unclear, circulating tumor DNA fragments tend to be shorter than cfDNA originating from normal tissues (28–30, 113, 114). It can be related to globally altered methylation and histone modifications [epigenetic changes that may alter the tightness of DNA wrapping around the nucleosomes (24, 115, 116)] or to aberrant mechanisms of DNA fragmentation in tumors and their microenvironments. Moreover, some researchers associate the observed difference with immune activity (114). Either way, cfDNA fragmentation features reflect massive epigenetic changes in tumor cells and may be considered as novel types of tumor markers (117, 118).

Several enzymes that are likely responsible for cfDNA fragmentation have been recently extensively studied in murine models (119–121). Generally, they could be attributed to three types based on localization (24). The first ones act in the cells of cfDNA origin during active cell death (e.g., apoptosis), with the caspase-activated DNase being one of the most widely known. Other nucleases cleave DNA during phagocytosis or in the extracellular space (e.g., deoxyribonuclease 1, deoxyribonuclease 1 like 3), and some secreted enzymes retain activity in the blood. Evidence suggests moderate sequence specificity of the enzymes acting in blood and apoptotic cells (119, 120), while tissue and macrophage-localized nucleases seem to introduce additional diversity to preferred ctDNA end sequences in cancer (122). As a result, cfDNA sampled from the bloodstream may bear the signs of consecutive exposure to a number of nucleases.

To date, several strategies have been proposed to apply these concepts for tumor detection. Cristiano et al. investigated cfDNA fragmentation in cancer patients at a whole genome scale (30). Their classifier predicted tumor types based on the ratios of longer and shorter fragments in the bins across the genome sequenced with low coverage. Analysis of cfDNA fragmentation focused on tumor-specific transcription factor binding sites revealed patterns that may reflect critical changes in tumor cells’ epigenetic regulation (117). Another consequence of non-random cfDNA fragmentation is an uneven distribution of the ends of fragments across the genome. It has been shown that the preferred DNA end coordinates may be characteristic of the tissue of origin (123). Moreover, accounting for the orientation of fragments may facilitate the detection of tissue-specific cfDNA fraction (124). The sequence specificity of nucleases involved in cfDNA formation can also be exploited to detect tumor presence. For instance, a biased distribution and increased diversity of sequence motifs were described in the ends of cfDNA fragments in patients with liver cancer (122). The technical loss of short (<100 bp) and degraded (nicked, partially single-stranded) DNA fragments during sample preparation for next-generation sequencing results in their underrepresentation in the final library. Similar issues in handling ancient DNA are addressed mostly by certain enzymes’ ability to ligate single-stranded templates (125, 126). Based on these developments and original ideas, novel methods are constantly proposed to increase the recovery of shorter cfDNA fragments enabling precise profiling of fragment size distributions (127–130).

## Conclusions and Prospects

**Figure 2:**
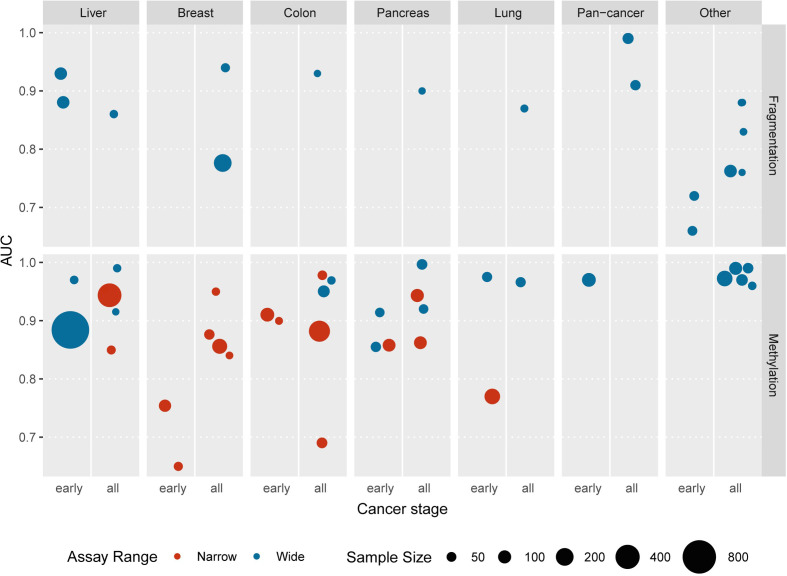
The recent studies reporting the application of epigenetic cfDNA assays for the detection of various tumor types are represented as areas under the ROC curves (AUCs) indicated in the respective publications. “Pan-cancer” category includes unified tests aimed at the detection of several cancer types; “other” category includes esophagus, bile duct, blood, central nervous system, gastric, kidney, ovarian, and urothelial cancers. The reported stages of cancers are grouped into two categories: “early” — pre-diagnosis or stage I or stage II and “all” — any other combination of stages or unspecified. The number of cancer patients involved in each study is plotted as the areas of the circles. Color represents the number of genomic loci included into each assay: red denotes narrow assays involving 20 loci or less, blue indicates whole-genome assays or larger targeted panels (more than 20 regions). Underlying data summarized in **Supplementary Table 1**.

The presence of tumor-derived DNA molecules in the plasma of cancer patients allows tumor detection and profiling with non-invasive blood tests. In practice, it is complicated by several biological factors that affect reproducibility and require ultrasensitive assays for reliable detection. Various ctDNA analysis strategies are optimal in different clinical scenarios due to the diversity of underlying biological and methodological foundations. Despite only a fraction of liquid biopsy capabilities being utilized in clinical cancer care by now, some prospects may be extrapolated. The detection of somatic mutations in ctDNA may reveal the genomic profile of the tumor, facilitating prognosis, response monitoring, and targeted therapy selection, making mutation-based ctDNA analysis techniques arguably the most widely adopted to date (131). MRD detection may also be based on tumor-specific mutations detection (132). With the development of novel targeted therapies and accumulation of the knowledge interconnecting clinical outcomes and genomic biomarkers, the practice of ctDNA mutation-based analysis will expand, supporting clinical decisions for more cancer types beyond lung, breast, gastric, and colon cancers. At the same time, rigorous attention should be given to interpreting mutations that may occur in normal tissues (133), especially during clonal hematopoiesis (134). Beyond the analysis of tumor-specific sequence alterations in ctDNA, epigenetic marks may be favorable for many applications due to their cell-type specificity and patterned nature. The landscape of available epigenetic-based cfDNA assays is represented mainly by cytosine methylation tests targeting a narrow set of well-established differentially methylated loci or more complex wide-range approaches that infer from ensembles of individual methylation markers [**Figure 2** and **Supplementary Table 1**; recently reviewed in detail in (135, 136)]. Recent extensive early detection efforts are based on the analysis of the broad panels of differentially methylated regions (51, 52), and this strategy may result in reliable screening tests. Analysis of methylation markers in cfDNA can also facilitate prognosis, recurrence monitoring, and management of cancers of unknown primary (137–140). The rising field of cfDNA fragmentomics has already yielded some promising approaches with comparable overall performance. Further developments in the field may include targeted fragmentation assays focused on the differentially fragmented regions and novel methods of deciphering epigenetic marks from fragment size distributions, end motifs, or new fragmentomic features. Although the fragmentation-based ctDNA analysis is still far from adoption for routine clinical use itself, it may be incorporated as an additional dimension to mutation-based or methylation-based liquid biopsy assays (28, 141). Furthermore, the possibility to improve tumor detection and characterization may lie in the simultaneous analysis of multiple marker types available from liquid biopsies, including proteins, circulating RNAs, tumor cells, and vesicles.

Cancer diagnostics may greatly benefit from the comprehensive characterization of hallmark events occurring in the early stages of tumorigenesis. As our understanding of these processes expands, future research in liquid biopsy may focus on identifying signs of premalignant growths’ progression in cell-free DNA. The interception of metastases is another crucial component of improving cancer management that can be further enhanced by liquid biopsy. We suppose that epigenetic-based approaches to the analysis of cfDNA features will play an increasingly important role in translating fundamental findings to clinical settings.

## Author Contributions

AK and DS wrote the main text. DS and NK conceptualized, planned, and supervised the preparation of the manuscript. KB, AK, and DS gathered data. DS and KB designed and prepared figures. NK and DS critically revised the manuscript. All authors contributed to the article and approved the submitted version.

## Funding

This study was supported by a grant from the Russian Science Foundation (project #20-75-10008).

## Conflict of Interest

The authors declare that the research was conducted in the absence of any commercial or financial relationships that could be construed as a potential conflict of interest.
