# Supplementary material for: The Detection of Cancer Epigenetic Traces in Cell-Free DNA
Source: Front Oncol. 2021 Apr 29;11:662094. doi: 10.3389/fonc.2021.662094 (PMC8118693; doi:10.3389/fonc.2021.662094)
Supplement: Supplementary file 2 [file DataSheet_1.docx]

**Supplementary Text**

Monte Carlo simulations were performed using R language. Input genome equivalents of cfDNA were selected from the range 303-30303 with the steps of 50. To further convert genome equivalents to ng of DNA we assumed the weight of a human haploid genome as ~3 pg. Tumor fractions in the samples were 0.001%, 0.01%, 0.1%. The number of alterations in the samples was 1, 5, and 10. At least 5 observations of any alteration were required to classify the sample as cancer positive. Simulations were performed for each combination of genome equivalents, tumor fractions, and the number of alterations listed above. The simulations included several steps:

1. The generation of a “blood sample” that contained 1000000 genomes for every alteration. The genomes within the generated “blood sample”, equal to tumor fraction, were marked as containing alteration.
2. The sampling of genome equivalent molecules from a generated “blood sample” for each alteration without replacing.
3. If the sum of sampled genomes with any alteration was ≥5, this sample was considered as cancer positive.
4. Each simulation was performed 10000 times and the percentage of cancer positives was calculated resulting in the probability of detection value.
